# The role of infectious disease consultations in the management of patients with fever in a long-term care facility

**DOI:** 10.1371/journal.pone.0291421

**Published:** 2023-09-08

**Authors:** Soo-youn Moon, Kyoung Ree Lim, Jun Seong Son

**Affiliations:** Division of Infectious Diseases, Department of Internal Medicine, Kyung Hee University College of Medicine, Kyung Hee University Hospital at Gangdong, Seoul, Korea; Shiraz University of Medical Sciences, ISLAMIC REPUBLIC OF IRAN

## Abstract

**Background:**

Infectious disease (ID) clinicians can provide essential services for febrile patients in tertiary hospitals. The aim of this study was to evaluate the role of ID consultations (IDC) in managing hospitalized patients with infections in an oriental medical hospital (OMH), which serves as a long-term care facility. To our knowledge, this is the first study on the role of IDCs in managing patients in an OMH.

**Methods:**

This retrospective study was conducted in an OMH in Seoul, Korea, from June 2006 to June 2013.

**Results:**

Among the 465 cases of hospital-acquired fever, 141 (30.3%) were referred for ID. The most common cause of fever was infection in both groups. The peak body temperature of the patient was higher in IDC group (38.8±0.6°C vs. 38.6±0.5°C, p<0.001). Crude mortality at 30 days (14.6% vs. 7.8%, p = 0.043) and infection-attributable mortality (15.3% vs. 6.7%, p = 0.039) were higher in the No-IDC group. Multivariable analysis showed that infection as the focus of fever (adjusted Odd ratio [aOR] 3.49, 95% confidence interval (CI) 1.64–7.44), underlying cancer (aOR 10.32, 95% CI 4.34–24.51,), and multiorgan dysfunction syndrome (aOR 15.68, 95% CI 2.06–119.08) were associated with increased 30-day mortality. Multivariate analysis showed that in patients with infectious fever, appropriate antibiotic therapy (aOR 0.19, 95% CI 0.05–0.76) was the only factor associated with decreased infection-attributable mortality while underlying cancer (aOR 7.80, 95% CI 2.555–23.807) and severe sepsis or septic shock at the onset of fever (aOR 10.15, 95% CI 1.00–102.85) were associated with increased infection-attributable mortality.

**Conclusion:**

Infection was the most common cause of fever in patients hospitalized for OMH. Infection as the focus of fever, underlying cancer, and MODS was associated with increased 30-day mortality in patients with nosocomial fever. Appropriate antibiotic therapy was associated with decreased infection-attributable mortality in patients with infectious fever.

## Introduction

Patients with advanced malignancies, other chronic illnesses, or older adults with limited mobility may need help performing basic daily activities. Many such people are cared for in long-term care facilities (LTCFs), including nursing hospitals. If they develop fever, a common symptom of infection, an infectious disease consultation (IDC) can be helpful, if available.

The role of IDCs has been described in several studies and is usually conducted at tertiary hospitals [1–3]. Few studies have focused on the role of IDCs in LTCFs, which focus primarily on reducing antimicrobial therapy [4, 5]. Patients staying in LTCFs can develop fever while being cared for in facilities. The etiology and management of fever acquired in LTCFs may differ from those acquired in acute care facilities, as patient needs and preferences differ [6].

In Korea, oriental medical hospitals (OMHs) play a role in providing medical services in the form of LTCFs, especially for those who want extra-medical care with oriental medicine treatment, including acupuncture, moxibustion and herbal medicine [7]. In Korea, oriental doctors can only perform oriental medical procedures and prescribe oriental herbal medicines. They cannot prescribe medications, including antimicrobial agents, or perform blood tests, X-rays, or other conventional diagnostic tests. Patients staying in OMHs need to consult doctors in medical hospitals for such tests and medications. Usually, fever is managed with herbal medicine in OMHs.

Most OMHs are not affiliated with university hospitals, where IDCs are available. Kyung Hee University Hospital at Gangdong, Seoul, Korea, comprises one medical hospital, one OMH, and one dental hospital. When patients in the OMH need medical attention, they can be referred to the medical hospital at any time. Most patients hospitalized in OMHs have advanced cancer or severe stroke, as in other LTCFs. A previous study found infection is the most common cause of hospital-acquired fever in OMHs [8].

There are some studies on the impact of IDC on infection or antimicrobial therapy. IDC improved the management and outcome of patients with bloodstream infection [9–11]. Early IDC is associated with lower mortality in patients with severe sepsis or septic shock [12]. IDC also improved antibiotic usage or antifungal agent usage [2, 13, 14] But as far as we know, there is no study addressing the impact of IDC on the management of nosocomial fever.

This study aimed to identify the role of IDCs in the management of patients with nosocomial fever in OMHs.

## Material and methods

This retrospective study was conducted at an OMH at a university medical institute in Seoul, Korea. The medical institute consists of a medical hospital, an OMH, and a dental hospital. The study protocol was approved by the institutional review board (IRB) of Kyung Hee University Hospital at Gangdong (IRB No. 2016-02-008). The IRB waived the requirement for informed consent from patients.

The electronic medical records of patients hospitalized in the OMH from June 1, 2006, to June 30, 2013, were retrospectively reviewed by two infectious disease (ID) specialists from April 2016. Patients aged 18 years and older were screened. Adult patients with an axillary body temperature ≥38°C after 48 hours of hospitalization were enrolled. Patients who were transferred from another OMH where they had been admitted for more than 48 hours and who developed a fever within 48 hours of hospitalization were also considered to have hospital-acquired fevers and were enrolled in the study. Patients were excluded if they were transferred from an acute medical hospital or LTCF or if the fever had started within 48 hours of hospitalization in the OMH.

Data on patient demographic characteristics, clinical features, laboratory data, and treatment history were extracted from the medical records with anonymization. Infection was defined using the US Centers for Disease Control and Prevention criteria [15]. Multiorgan dysfunction syndrome (MODS), severe sepsis, and septic shock were defined using criteria proposed by the Consensus Conference Committee of the American College of Chest Physicians and the Society of Critical Care Medicine [16]. The severity of the underlying illness was classified according to the McCabe score [17]. Defervescence was defined as a peak body temperature below 37.3°C for more than 2 consecutive days.

The antibiotic therapy was considered appropriate if the isolated organism was susceptible to the antibiotic used or the antibiotic was recommended by the treatment guidelines.

SPSS for Windows version 11.0 (SPSS Inc., Chicago, IL, USA) was used for the statistical analysis. Student’s t-test, Mann-Whitney U test, and chi-square tests were used for univariate analysis. Multivariable analysis was performed using logistic regression. Two-sided p values <0.05 were considered significant.

## Results and discussion

A total of 11207 adult patients were hospitalized in the OMH during the study period. Four hundred sixty-five cases (4.1%) of nosocomial fever were identified. One hundred and forty-one patients (30.3%) had an IDC for evaluation and management of fever (IDC group), and 324 patients (69.7%) did not have an IDC (No-IDC group). The mean age of the patients was similar, and approximately half of the patients in both groups were male (Table 1). More patients in the IDC group had underlying chronic illnesses, such as diabetes mellitus (DM), hypertension, or a past cerebrovascular accident, whereas more patients in the No-IDC group had underlying malignancies. More patients in the IDC group had a history of previous hospital admission or antibiotic treatment. The peak body temperature of patients in the IDC group was higher than that of patients in the No-IDC group (38.8±0.6°C vs. 38.6±0.5°C, p<0.001). The most common cause of fever was infection in both groups, with a significantly higher proportion in the IDC group (77.3% vs. 46.9%, p<0.001). A larger proportion of patients in the IDC group was treated with antibiotics (88.7% vs. 45.7%, p<0.001). Most patients in both groups experienced defervescence, but the duration of fever was longer in the IDC group (median 3 days, range 1–38 days). The mortality within 30 days after the onset of fever was higher in the No-IDC group (14.6% vs. 7.8%, p = 0.043).

**Table 1:** Characteristics of patients with fever according to whether they received an infectious disease consultation (n = 465).

	No IDC (n = 324)	IDC (n = 141)	p value
Age* (years), mean±SD	60.2±15.5	63.3±14.6	0.043
Male, n (%)	159 (49.1)	67 (47.5)	0.758
**Underlying disease, n (%)**			
Diabetes mellitus*	74 (22.8)	47 (33.3)	0.018
Hypertension*	107 (33.0)	84 (59.6)	<0.001
Cerebrovascular accident*	113 (34.9)	89 (63.1)	<0.001
Cancer*	175 (54.0)	34 (24.1)	<0.001
McCabe score (ultimately or rapidly fatal)	323 (99.7)	140 (99.3)	0.544
**Indwelling catheter, n (%)**			
Central venous catheter	40 (12.4)	22 (15.6)	0.342
Foley catheter*	51 (15.7)	35 (24.8)	0.020
Percutaneous draining catheter	36 (11.1)	24 (17.0)	0.081
VP shunt	9 (2.8)	4 (2.8)	0.972
**Previous treatment, n (%)**			
Previous surgery	39 (12.0)	26 (18.4)	0.067
Previous admission	234 (72.2)	115 (81.6)	0.032
Previous antibiotics*	55 (17.0)	51 (36.2)	<0.001
**Presentation of fever**			
Peak body temperature* (°C), mean±SD	38.6±0.5	38.8±0.6	<0.001
MODS*, n (%)	245 (75.6)	124 (87.9)	0.002
**Fever focus, n (%)**			
Infection*	152 (46.9)	109 (77.3)	<0.001
Non-infection*	135 (41.7)	23 (16.3)	<0.001
Unknown*	31 (9.6)	2 (1.4)	0.001
**Treatment for fever, n (%)**			
Antibiotics*	148 (45.7)	125 (88.7)	<0.001
Antipyretics	68 (21.0)	39 (27.7)	0.116
Surgery	7 (2.2)	0 (0.0)	0.213
**Outcome**			
Defervescence, n (%)	288 (88.9)	130 (92.2)	0.276
Duration of fever* (days), median (IQR)	2 (1–18)	3 (1–38)	<0.001
Mortality at 30 days*, n (%)	41 (14.6)	9 (7.8)	0.043

IDC, infectious disease consultation; IQR, interquartile range; MODS, multiorgan dysfunction syndrome; SD, standard deviation; VP, ventriculoperitoneal

The percentages shown in the table are unweighted.

*: those with significant p values

Among patients with infectious fever, the severity of underlying chronic illnesses did not differ significantly between the groups according to the McCabe score. Cultures were performed more frequently in the IDC group (96.3% vs. 80.3%, p<0.001) (Table 2). Respiratory tract infection was more common in the No-IDC group (57.9% vs. 25.7%, p<0.001), whereas urinary tract infection was more common in the IDC group (49.5% vs. 22.4%, p<0.001). Patients with severe sepsis or septic shock were more likely to have an IDC and were more likely to have an infection of bacterial origin than those in the No-IDC group (95.4% vs. 76.3%, p<0.001) (S1 Table). Most of the patients were treated with antibiotics for fever, with a higher proportion being treated in the IDC group (96.3% vs. 71.7%, p<0.001). A higher proportion of the IDC group received appropriate antibiotic therapy (96.3% vs. 80.9%, p<0.001). Both the crude 30-day mortality and infection-attributable mortality rates were higher in the No-IDC group (19.9% and 15.3%, respectively).

**Table 2:** Characteristics of patients with infectious fever according to whether they received an infectious disease consultation (n = 261).

	No IDC (N = 152)	IDC (N = 109)	p value
Age (years), mean±SD	62.7±15.5	64.1±14.9	0.805
Male, n (%)	74 (48.7)	49 (45.0)	0.552
**Underlying disease, n (%)**			
DM	39 (25.7)	32 (29.4)	0.508
HTN*	53 (34.9)	68 (62.4)	<0.001
Cerebrovascular accident*	61 (40.1)	69 (63.3)	<0.001
Cancer*	69 (45.4)	26 (23.9)	<0.001
McCabe score (ultimately or rapidly fatal)	152 (100.0)	108 (99.1)	0.418
**Indwelling catheter, n (%)**			
Central venous catheter	21 (13.9)	19 (17.4)	0.424
Foley catheter	29 (19.1)	27 (24.8)	0.269
Percutaneous draining catheter	20 (13.2)	21 (19.3)	0.181
VP shunt	6 (4.0)	3 (2.8)	0.602
**Previous treatment, n (%)**			
Previous surgery	17 (11.2)	21 (19.3)	0.068
Previous admission	107 (70.4)	88 (80.7)	0.058
Previous antibiotics*	32 (21.1)	43 (39.5)	0.001
**Presentation of fever**			
Peak body temperature (°C), mean±SD	38.7±0.6	38.8±0.6	0.835
Severe sepsis or septic shock, n (%)	126 (82.9)	97 (89.0)	0.144
Culture study*, n (%)	122 (80.3)	105 (96.3)	<0.001
**Infection focus, n (%)**			
Respiratory tract infection*	88 (57.9)	28 (25.7)	<0.001
Urinary tract infection*	34 (22.4)	54 (49.5)	<0.001
Biliary tract infection	8 (5.3)	7 (6.4)	0.692
Intra-abdominal infection	7 (4.6)	4 (3.7)	0.711
CNS infection	0 (0.0)	2 (1.8)	0.094
Bone and joint infection	0 (0.0)	1(0.9)	0.237
Catheter-related infection	2 (1.3)	4 (3.7)	0.211
Skin and soft tissue infection	7 (4.6)	9 (8.3)	0.225
Other	8 (5.3)	5 (4.6)	0.379
**Treatment for fever**			
Antibiotics*, n (%)	109 (71.7)	105 (96.3)	<0.001
Duration of antibiotic administration (days), median (IQR)	10 (1–38)	21 (1–46)	0.289
Appropriate antibiotic therapy*, n (%)	123 (80.9)	105 (96.3)	<0.001
Antipyretics, n (%)	40 (26.3)	28 (25.7)	0.909
Surgery, n (%)	4 (2.6)	0 (0.0)	0.233
**Outcome**			
Defervescence, n (%)	140 (92.1)	102 (93.6)	0.652
Duration of fever (days) *, median (IQR)	2 (1–18)	3 (1–38)	0.002
Mortality at 30 days*, n (%)	26 (19.9)	9 (10.0)	0.049
Infection-related death*, n (%)	19 (15.3)	6 (6.7)	0.039

CNS, central nervous system; DM, diabetes mellitus; HTN, hypertension; IDC, infectious disease consultation; IQR, interquartile range; SD, standard deviation; VP, ventriculoperitoneal

The percentages shown in the table are unweighted.

*: those with significant p values

Multivariable logistic regression showed that in patients with fever, infection (adjusted odds ratio [aOR] 3.49, 95% confidence interval [CI] 1.64–7.44), MODS at the onset of fever (aOR 15.68, 95% CI 2.06–119.08,), and cancer (aOR 10.32, 95% CI 4.34–24.51,) were associated with an increased odds of 30-day mortality (Table 3). Patients in the IDC group had lower mortality (aOR 0.53, 95% CI 0.22–1.26), but the decreased odds was not statistically significant (p = 0.152).

**Table 3:** Multivariable logistic regression analysis of risk factors for mortality in patients with fever.

	Odds ratio (95% confidence interval)	p value
Infection	3.49 (1.64–7.44)	0.001
MODS	15.68 (2.06–119.08)	0.008
Cancer	10.32 (4.34–24.51)	<0.001
BT ≥38.7°C	0.80 (0.41–1.58)	0.526

BT, body temperature; MODS, multiorgan dysfunction syndrome

In patients with infectious fever, underlying cancer (aOR 7.80, 95% CI 2.56–23.81) and severe sepsis or septic shock at the onset of fever (aOR 10.15, 95% CI 1.00–102.85) were factors associated with an increased odds of infection-attributable mortality. Appropriate antibiotic therapy (aOR 0.19, 95% CI 0.05–0.76) was the only factor associated with decreased odds of infection-attributable mortality (Table 4).

**Table 4:** Multivariable logistic regression analysis of risk factors for infection-related mortality in patients with infectious fever.

	Odds ratio (95% confidence interval)	p value
ID consultation	1.08 (0.34–3.43)	0.890
Respiratory tract infection	2.84 (0.83–9.75)	0.097
UTI	6.70 (0.68–65.85)	0.103
Cancer	7.80 (2.56–23.81)	<0.001
BT ≥39°C	1.56 (0.52–4.73)	0.432
Severe sepsis or septic shock	10.15 (1.00–102.85)	0.050
Appropriate antibiotic therapy	0.19 (0.05–0.76)	0.019

BT, body temperature; ID, infectious disease; UTI, urinary tract infection

In Korea, patients needing supportive care for chronic medical conditions, such as cerebral infarction or terminal cancer, are hospitalized for extra-medical care with oriental medical treatment, including acupuncture and herbal medicine, whose underlying characteristics are similar to those in LTCFs [7]. In this study, among 402 cases, 133 patients (33.1%) consulted ID specialists to evaluate the cause and management of fever. Only a few data are available on the role of IDC in patient care in Korea [18, 19], which are mainly focused on patients in acute care facilities.

To the best of our knowledge, there are no data available on the role of IDC in nosocomial fever in patients hospitalized in OMHs or LTCFs in Korea. There are some data on IDC’s role in acute care facilities, such as ICUs or tertiary hospitals. Madeline et al [12] reported that early IDC was associated with a 40% risk reduction in in-hospital mortality among patients receiving a severe sepsis/septic shock bundle. A study by Kim and colleagues [18] showed that IDC was associated with improved 28-day survival after blood draw for culture. Most studies on the role of ID consultation in LTCFs from other countries have focused on the appropriate use of antibiotics [13, 14, 20].

The 30-day mortality rate of patients with nosocomial fever was higher in the No-IDC group. This may be due to the higher proportion of patients with malignancies in the No-IDC group. Multivariable logistic regression revealed that malignancy was an independent factor for 30-day mortality in patients with nosocomial fever. Similar to the findings of a study on IDCs for multidrug-resistant bacteremia by Kim et al. [18], in this study, patients with malignancies were not more likely to have an IDC. This may be attributable to Do-Not-Resuscitate orders and refusal of further evaluation and treatment in patients with advanced malignancies.

In this study, the most common cause of fever was infection in both groups, similar to the findings of other studies [19, 21, 22]. The infection-attributable mortality rate of patients with nosocomial infectious fever was higher in the No-IDC group. The duration of fever and antibiotic therapy were longer in the IDC group than in the No-IDC group. This might have been due to the greater severity of the infection because patients with severe sepsis or septic shock and were more likely to have an infection of bacterial origin and would be consulted to ID more often. More patients in the IDC group had culture study, which can be helpful in deciding appropriate antibiotic therapy. The antibiotic therapy was more appropriate in the IDC group. There are some data showing that IDC is related to more appropriate antibiotic therapy [13, 14, 23, 24]. A study by Jump et al. showed that IDC in LTCF can be an effective means to bring subspecialty care to LTCF residents [24]. Multivariable analysis showed that underlying malignancy, severe sepsis, and septic shock at the onset of fever were associated with infection-attributable mortality. Although IDC was not associated with an improved outcome in this study, appropriate antibiotic therapy can improve the outcome. Previous studies have shown that IDCs are associated with an increased likelihood of receiving appropriate empirical antimicrobial treatment, which leads to improved survival in patients with bacteremia or candidemia [9, 25]. Timely and appropriate antibiotic treatment can also reduce length of hospital stay, and treatment failure [26, 27].

This study has some limitations. First, it was a retrospective study performed in an OMH. Culture and imaging studies were not performed for every patient with fever. Therefore, some patients with infection might not have been identified and might have been classified as having a fever of unknown origin, and some patients identified as having infection may have had fevers of non-infectious origin. Second, this study was conducted at a single facility. The study OMH treated many patients with advanced cancer or cerebrovascular events, and patients had more ready access to IDCs than in other OMHs, limiting the generalizability of the results to other OMHs. Therefore, further studies are necessary.

## Conclusion

Infection was the most common cause of nosocomial fever in patients hospitalized in the study OMH. Patients in the IDC group were more likely to have bacterial infections and had a longer duration of antimicrobial therapy Although there was no statistical significance due to small number of cases, IDC could be associated with reduced mortality in febrile patients with more comorbidities, fevers of infectious origin, severe sepsis or septic shock, or MODS.

## Supporting information

S1 Checklist STROBE statement—checklist of items that should be included in reports of observational studies. (DOCX)

S1 Table Organisms of infection in patients with infectious fever (n = 261). (DOCX)

S2 Table Laboratory test results of patients with fever (n = 465). (DOCX)

S3 Table Laboratory test results of patients with infectious fever (n = 261). (DOCX)
