# Supplementary material for: The role of infectious disease consultations in the management of patients with fever in a long-term care facility
Source: PLoS One. 2023 Sep 8;18(9):e0291421. doi: 10.1371/journal.pone.0291421 (PMC10491299; doi:10.1371/journal.pone.0291421)
Supplement: S1 Table — (DOCX) [file pone.0291421.s002.docx]

S1 Table. Organisms of infection in patients with infectious fever (n=261)

| **Organisms** | No IDC (n=152) | IDC (n=109) | p value |
| --- | --- | --- | --- |
| Bacteria | 116 (76.3) | 104 (95.4) | <0.001 |
| Virus | 33 (21.7) | 5 (4.6) | 0.016 |
| Fungus | 3 (2.0) | 0 (0.0) | 0.252 |
