# Supplementary material for: The role of infectious disease consultations in the management of patients with fever in a long-term care facility
Source: PLoS One. 2023 Sep 8;18(9):e0291421. doi: 10.1371/journal.pone.0291421 (PMC10491299; doi:10.1371/journal.pone.0291421)
Supplement: S2 Table — (DOCX) [file pone.0291421.s003.docx]

S2 Table. Laboratory test results of patients with fever (n=465)

|  | No IDC (n=324) | IDC (n=141) | p value |
| --- | --- | --- | --- |
| WBC | 8700 (800-28200) | 10500 (100-32500) | 0.018 |
| C-reactive protein | 5.6 (0.0-27.2) | 6 (0.0-40.0) | 0.816 |
| AST | 25 (8-382) | 24 (10-150) | 0.053 |
| ALT | 21 (5-333) | 22 (4-147) | 0.441 |
| BUN | 14 (4-51) | 13 (2-55) | 0.716 |
| Cr | 0.8 (0.4-6.2) | 1 (0.1-4.0) | 0.456 |
