# Supplementary material for: The role of infectious disease consultations in the management of patients with fever in a long-term care facility
Source: PLoS One. 2023 Sep 8;18(9):e0291421. doi: 10.1371/journal.pone.0291421 (PMC10491299; doi:10.1371/journal.pone.0291421)
Supplement: S3 Table — (DOCX) [file pone.0291421.s004.docx]

S3 Table, Laboratory test results of patients with infectious fever (n= 261)

|  | No IDC (n=152) | IDC (n=109) | p value |
| --- | --- | --- | --- |
| WBC | 10600 (900-28200) | 10600 (2600 – 32500) | 0.585 |
| C-reactive protein | 8 (0-33) | 6 (0-40) | 0.110 |
| AST | 24 (8-296) | 23 (10-115) | 0.173 |
| ALT | 19 (6-239) | 20 (4-106) | 0.895 |
| BUN | 14 (0.0-62.0) | 13 (5-55) | 0.726 |
| Cr | 1 (0.1-6.0) | 1 (0.1-4.0) | 0.495 |
